# The Allosteric HIV-1 Integrase Inhibitor BI-D Affects Virion Maturation but Does Not Influence Packaging of a Functional RNA Genome

**DOI:** 10.1371/journal.pone.0103552

**Published:** 2014-07-29

**Authors:** Nikki van Bel, Yme van der Velden, Damien Bonnard, Erwann Le Rouzic, Atze T. Das, Richard Benarous, Ben Berkhout

**Affiliations:** 1 Laboratory of Experimental Virology, Department of Medical Microbiology, Center for Infection and Immunity Amsterdam (CINIMA), Academic Medical Center, University of Amsterdam, Amsterdam, the Netherlands; 2 Biodim Mutabilis, Romainville, France; Queensland Institute of Medical Research, Australia

## Abstract

The viral integrase (IN) is an essential protein for HIV-1 replication. IN inserts the viral dsDNA into the host chromosome, thereby aided by the cellular co-factor LEDGF/p75. Recently a new class of integrase inhibitors was described: allosteric IN inhibitors (ALLINIs). Although designed to interfere with the IN-LEDGF/p75 interaction to block HIV DNA integration during the early phase of HIV-1 replication, the major impact was surprisingly found on the process of virus maturation during the late phase, causing a reverse transcription defect upon infection of target cells. Virus particles produced in the presence of an ALLINI are misformed with the ribonucleoprotein located outside the virus core. Virus assembly and maturation are highly orchestrated and regulated processes in which several viral proteins and RNA molecules closely interact. It is therefore of interest to study whether ALLINIs have unpredicted pleiotropic effects on these RNA-related processes. We confirm that the ALLINI BI-D inhibits virus replication and that the produced virus is non-infectious. Furthermore, we show that the wild-type level of HIV-1 genomic RNA is packaged in virions and these genomes are in a dimeric state. The tRNA^lys3^ primer for reverse transcription was properly placed on this genomic RNA and could be extended *ex vivo*. In addition, the packaged reverse transcriptase enzyme was fully active when extracted from virions. As the RNA and enzyme components for reverse transcription are properly present in virions produced in the presence of BI-D, the inhibition of reverse transcription is likely to reflect the mislocalization of the components in the aberrant virus particle.

## Introduction

Current therapy for HIV-1 infected individuals consists of a combination of antiretroviral drugs that target essential steps of the virus replication cycle: virus entry into the host cell and the subsequent processes executed by the viral enzymes reverse transcriptase (RT), integrase (IN) and protease (PR). The most recently approved drugs Raltegravir [1], Elvitegravir [2] and Dolutegravir [3] block HIV-1 DNA integration into the cellular genome by binding to the IN active site and belong to the class of strand transfer inhibitors (INSTIs).

The HIV-1 pre-integration complex is tethered to the host chromosome via the cellular co-factor lens epithelium-derived growth factor (LEDGF/p75) [4]. LEDGF/p75 binds chromatin via the N-terminal domain [5] and the C-terminal IN binding domain (IBD) recognizes the cleft formed at the IN dimeric interface [6], [7]. Because of the rapid emergence of cross-resistance among INSTI drugs, new IN-inhibitors were designed that prevent this IN-LEDGF/p75 interaction: LEDGINs [8], tBPQAs [9], INLAIs [10], NCINIs [9], [11], [12] or, as we will call them, ALLINIs [13]. ALLINIs are allosteric inhibitors that cause IN inactivation by triggering its multimerization in the absence of viral DNA [9]–[11], [13]–[16]. Prevention of the LEDGF/p75-IN interaction accounts for the “early” block of HIV-1 replication, but it was recently described that the major impact of ALLINIs is exerted in the “late” phase, on IN multimerization and in particular virion maturation [10], [11], [15], [16].

During virus maturation the virion morphology changes from a core, in which the ribonucleoprotein (RNP) is located in a ring-shape at the virus membrane, to a virus particle in which the RNP is condensed into a conical core formed by the capsid protein. Virions produced in the presence of ALLINIs are non-infectious and contain aberrant cores as shown by electron microscopy [11], [15], [16]. The electron-dense RNP that includes the RNA genome is mislocalized outside the core, which otherwise remains intact. These virions were able to infect target cells, but the subsequent reverse transcription process was found to be blocked [11], [16]. Because ALLINIs bind to IN, it was proposed that the drugs interact with the IN domain as part of the precursor Gag-Pol polyprotein during virus production, thus affecting virion assembly and maturation.

Virus assembly and maturation are highly orchestrated and regulated processes in which several protein and RNA partners come together to create an infectious virion particle that is able to execute the complex process of reverse transcription of the RNA genome upon infection of a new cell. The Gag and Gag-Pol polyproteins are selectively packaged into nascent virions together with the dimeric RNA genome and the tRNA^lys3^ primer for reverse transcription [17]. After budding from the cell surface, virus particles mature to become fully infectious, which involves cleavage of the Gag and Gag-Pol polyproteins by PR to generate the individual structural subunits and to activate the enzymes. PR-mediated cleavage occurs in a sequential order with different processing rates for individual cleavage sites, the first step being the autocatalytic release of PR from the Gag-Pol precursor [18], [19].

The different RNA steps are interwoven with protein events during virion maturation. Several examples will be presented. The Gag precursor packages a labile genomic RNA dimer through interaction of the nucleocapsid (NC) domain with the structured Ψ signal in the 5 leader region of the HIV-1 RNA [20]–[28]. PR-mediated virion maturation is linked to stabilization of this RNA dimer. Mutation of the PR cleavage sites in Gag severely affects virus maturation and especially cleavage at the p2-NC site in Gag is essential for proper RNA dimer maturation [29]–[31]. Mature NC protein acts as chaperone in RNA dimer stabilization [30], [32]–[34] and IN has also been suggested to influence RNA dimerization [35]. Pol-processing mutants display an altered RNA dimerization profile, although IN multimerization mutants were not affected [36]. A SELEX study, in which RNA ligands that bind with high affinity to IN were identified, suggested an interaction between IN protein and HIV-1 RNA. A possible interference of BI-D with this interaction may cause more direct HIV-1 RNA effects [37]. These pleiotropic effects of the Gag-Pol polyprotein underscore the complex RNA-protein interconnections during HIV-1 virion assembly and maturation.

Reverse transcription uses a tRNA^lys3^ primer that is selectively packaged in newly assembled virions by the aminoacyl tRNA synthetases LysRS (reviewed in [38]). The tRNA annealing complex is formed by interaction with the viral RNA-Gag-GagPol complex. LysRS specifically recognizes Gag [39] and tRNA^lys3^ binds to the RT domain in the Gag-Pol polyprotein [40], [41]. tRNA^lys3^ is subsequently annealed to the HIV-1 RNA genome, facilitated by Gag. Upon processing of the viral polyproteins, the mature NC protein reinforces the tRNA^lys3^ interaction with the HIV-1 genome such that it can be extended by RT [42], [43] (and reviewed in [44]).

Subsequent RNA steps are also linked to virion maturation events. Certain IN mutants have a profound effect on reverse transcription, but do not affect the RT activity in viral lysates, suggesting an exclusive impact in the context of the virion particle. In addition, IN mutant viruses can be affected in a variety of processes, including integration, assembly and viral core morphology [45]–[48]. These combined results indicate that protein and RNA processing are intricately linked during virion assembly and maturation. Combining such potential RNA effects with the early and late effects of ALLINIs, this could be the first drug with three modes of action, thus providing a combinational therapy in a single drug. This sets the stage for testing the impact of ALLINIs, which grossly affect virion morphology, on the many RNA processes involved.

## Materials and Methods

### Cell culture

SupT1 T cells were cultured in advanced RPMI 1640 medium (Gibco) supplemented with 1% (v/v) heat-inactivated fetal bovine serum (FBS, Gibco), 2 mM L-glutamine (Gibco), 15 µg/ml streptomycin and 15 units/ml penicillin at 37°C and 5% CO_2_. Human embryonic kidney (HEK) 293T cells were grown in DMEM (Gibco) supplemented with 10% (v/v) heat-inactivated FBS (Gibco) and 1x minimum essential medium non-essential amino acids (MEM NEAA, Gibco) at 37°C and 5% CO_2_.

### BI-D

Integrase-inhibitor BI-D was prepared as described in [10] and in patent application ([49], according to example 41). BI-D was dissolved in DMSO to generate a stock solution of 10 mM and stored in aliquots at –20°C. BI-D was added to the culture medium at a final concentration of 700 nM (5x EC_50_) when indicated. The equivalent volume of DMSO was added to control cultures (final concentration: 0.035%).

### Virus production and replication

293T cells were seeded in T75 culture flasks, cultured to 50–70% confluency and transfected with 20 µg pLAI DNA plasmid that encodes the wt HIV-1 LAI isolate [50] using Lipofectamine 2000 (Invitrogen). BI-D was added at 6 h after transfection. The culture supernatant was harvested at 48 h after transfection and used as virus stock or for viral RNA isolation. The CA-p24 level was measured by enzyme-linked immunosorbent assay (ELISA) as described previously [51]. SupT1 T cells (5×10^6^ cells in 5 ml) were infected with the HIV-1 LAI virus stocks (equivalent of 1 ng CA-p24). When indicated, the culture was split and BI-D or DMSO was added. Viral spread was monitored by measuring the CA-p24 level in the virus culture medium every 1–2 days.

### Viral RNA isolation

Virus produced by 293T cells with or without BI-D was pelleted by ultracentrifugation over a 20% sucrose cushion in phosphate buffered saline at 32,000 rpm (175,000×g) for 2 h at 4°C in a Beckman SW32 Ti rotor. The pellet was resuspended in 400 µl lysis buffer (50 mM Tris-HCl pH 7.4, 10 mM EDTA, 100 mM NaCl and 1% SDS) and a sample was taken for CA-p24 measurement. Virions were lysed at 37°C for 30 min by addition of proteinase K (final concentration 100 µg/ml). Viral RNA was extracted twice with phenol-chloroform-isoamylalcohol (25:24:1) at 4°C. The sample was split in two parts, ethanol-precipitated and washed with 70% ethanol. RNA for primer extension was resuspended in 10 µl TE buffer (10 mM Tris-HCl pH 8.0, 1 mM EDTA) and stored at –80°C. RNA for northern blotting was resuspended in TN buffer (10 mM Tris-HCl pH 7.5, 100 mM NaCl) and treated with DNase (Ambion) for 60 min at 37°C. After incubation, the RNA was extracted once with phenol-chloroform-isoamylalcohol (25:24:1). The RNA was ethanol-precipitated with GlycoBlue (Ambion) as carrier and washed with 70% ethanol. The RNA pellet was resuspended in 40 µl TENS buffer (10 mM Tris-HCl pH 7.5, 1 mM EDTA, 100 mM NaCl, 1% SDS), aliquotted and stored at –80°C.

### Northern blot analysis

Non-denaturing and denaturing northern blot analysis was performed on viral RNA isolated from equal amounts of virions (equivalent of 250 ng CA-p24). For the non-denaturing northern blot, RNA was mixed with non-denaturing sample buffer (30% glycerol, 0.25% bromophenol blue dye) and analyzed by electrophoresis on a 0.9% agarose gel in 1x TBE buffer at 72 V, 4°C for 6 h. For the denaturing northern blot, viral RNA was mixed with denaturing loading dye (final concentration: 40 mM MOPS pH 7.0, 10 mM sodium acetate, 5% formaldehyde, 0.05 mg/ml ethidium bromide, 0.5 mg/ml orange G, 7 g/ml sucrose) and electrophoresed on a 0.9% agarose gel in MOPS buffer (40 mM MOPS, 10 mM sodium acetate pH 7.0) with 7% formaldehyde at 100 V for 4 h. The gel for non-denaturing northern blotting was soaked in 10% formaldehyde at 65°C for 30 min before blotting. For both northern blots, the RNA was transferred overnight by capillary force onto a positively charged nylon membrane (Roche) using 20x SSC (3.0 M NaCl, 0.3 M sodium citrate pH 7.0). An UV crosslinker (Stratagene) was used to cross-link the RNA to the membrane. The membrane was incubated for 1 h in ULTRAhyb (Ambion) at 55°C. The probe, consisting of a 1014-bp DNA fragment covering the Nef, U3 and R regions of the pLAI plasmid (positions 8770–9784, relative to the transcriptional start site at +1), was labeled with ^32^P by random-primed labeling (High Prime DNA labeling kit; Roche Diagnostics) using α^32^P-CTP (0.33 MBq/µl, Perkin-Elmer). The probe was added to the prehybridized membrane, followed by hybridization for 16 h at 55°C, after which the membrane was extensively washed. Quantification was performed using a phosphorimager (Amersham Biosciences) and the ImageQuant software package. To determine the thermal stability of the HIV-1 RNA dimer, viral RNA (equivalent of 250 ng CA-p24) in 10 µl TENS buffer was incubated at increasing temperatures (40–60°C) for 10 min before the RNA was mixed with 5 µl sample buffer (30% glycerol with 0.25% bromophenol blue dye) and analyzed on a non-denaturing northern blot. The Tm was calculated as the temperature at which 50% of the RNA dimer was melted into faster migrating RNA forms.

### tRNA and CN1 primer extension assay

Viral RNA (equivalent of 50 ng CA-p24) in 12 µl buffer (83 mM Tris-HCl pH 7.5, 125 mM KCl) was either used directly for tRNA^lys3^ extension or was mixed with primer CN1 (GGTCTGAGGGATCTCTAGTTACCAGAGTC, complementary to nucleotides 123–151 of LAI RNA), heated at 85°C for 2 min, at 65°C for 10 min, followed by slow-cooling to room temperature over 1 h to allow primer annealing. 6 µl 3x RT buffer (9 mM MgCl_2_, 30 mM DTT, 150 µg/ml actinomycin D, 30 µM dCTP, 30 µM dGTP, 30 µM dTTP and 1.5 µM dATP [Thermo-Scientific], 0.3 µl α32P-dATP [0.33 MBq/µl, Perkin-Elmer], 22 nM HIV-1 RT [2.5 U per sample; p51/p66 heterodimer; kindly provided by D. Stammers, Glaxo Wellcome Research Laboratories, MRC AIDS reagent project] was added to the tRNA^lys3^ and CN1 extension samples. The mixture was incubated at 37°C for 30 min to extend the naturally associated tRNA^lys3^ primer or the annealed CN1 DNA primer. The cDNA was precipitated in 25 mM EDTA, 0.3 M NaAc pH 5.2 and 80% EtOH at –20°C. cDNA pellets were washed with 70% ethanol and dissolved in gel-loading buffer II (Ambion). The cDNA was analyzed on a denaturing 6% polyacylamide-urea sequencing gel and bands were quantified using a phosphorimager (Amersham Biosciences) and ImageQuant software.

### RT activity assay

To measure RT activity, we used a real-time qPCR-based RT assay [52], [53]. HIV-1 LAI virus produced in the presence or absence of BI-D was diluted 1:10 in RT dilution buffer B (20 mM Tris-HCl pH 7.5, 50 mM KCl, 0.25 mM EDTA pH 8.0, 0.2 mM DTT, 0.025% Triton X-100, 50% glycerol) and 4 µl diluted virus was mixed with 6 µl RT mix (10 mM Tris-Cl pH 8.3, 50 mM KCl, 5 mM MgCl_2_, 0.0035% Triton X-100, 0.2 mM dNTPs, 2 mM DTT, 36 nM 3 primer A [GCCTTAGCAGTGCCCTGTCT], 8 units RNAsin [Roche] and 120 ng MS2 RNA [Roche]). AMV-RT (New England Biolabs) diluted in RT dilution buffer B was used to generate a standard curve. The amount of MS2 cDNA formed at 37°C for 4 h was quantitated in a TaqMan-PCR (AbiPrism7000, Applied Biosystems). 10 µl cDNA was mixed with 40 µl PCR-mix (final concentration: 0.8x Platinum Taq PCR buffer [Invitrogen], 2.5 mM MgCl_2_, 1x Rox Reference Dye [Invitrogen], 0.16 mM dNTPs, 0.51 µM 5 primer B [AACATGCTCGAGGGCCTTA], 0.51 µM 3 primer A, 0.15 µM MS2-probe [5 FAM-CCCGTGGGATGCTCCTACATGTCA-3 TAMRA], 1.25 U PlatinumTaq [Invitrogen]). The following PCR-scheme was used: 15 min at 37°C, 10 min at 95°C and 50 cycles of 15 sec 95°C, 15 sec at 56°C and 45 sec at 60°C. In each PCR-cycle the amount of DNA formed was measured.

## Results

### HIV-1 inhibition by the ALLINI BI-D

**Figure 1:**
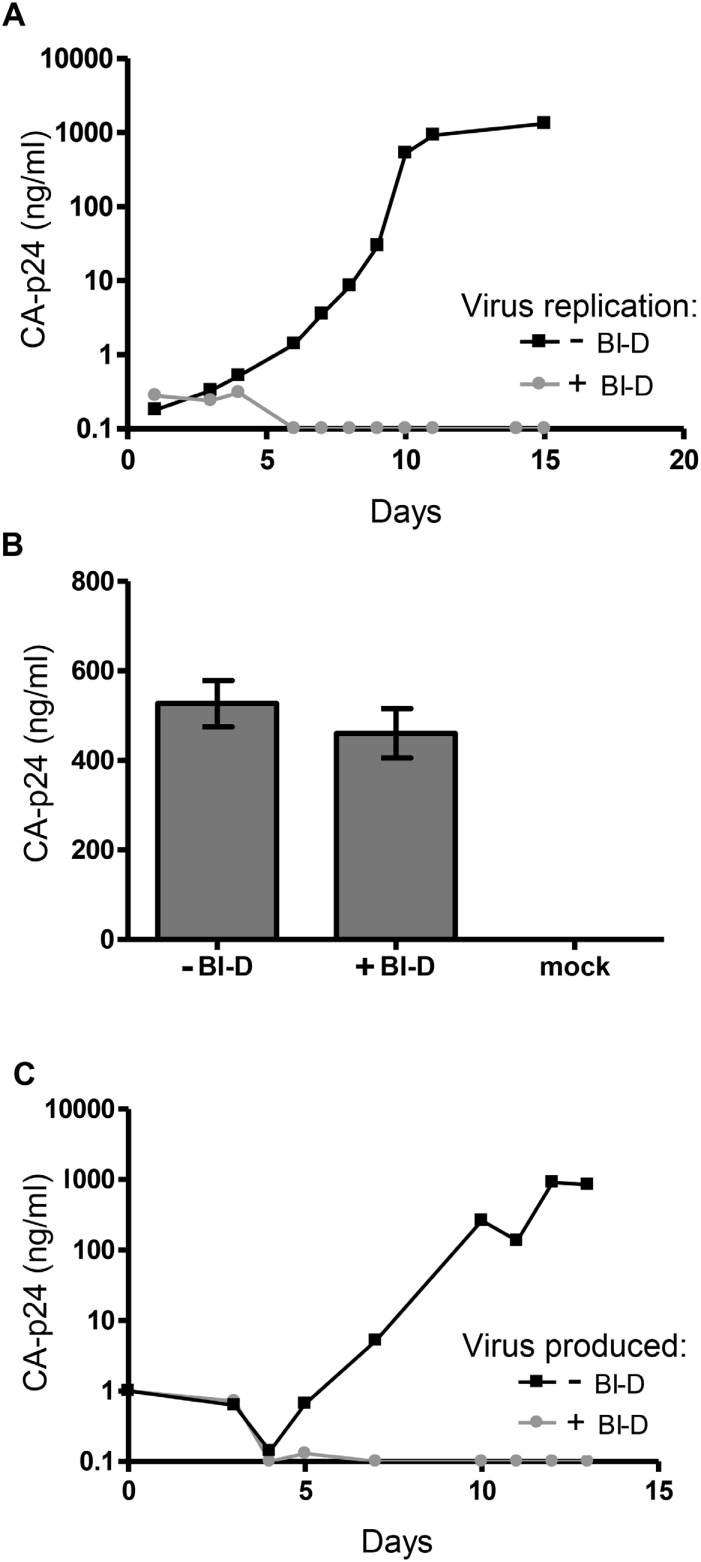
Impact of BI-D on HIV-1 replication and production. A. SupT1 cells were infected with HIV-1 LAI and cultured in the absence or presence of BI-D (700 nM, 5x EC_50_). B. 293T cells were transfected with HIV-1 LAI plasmid, cultured with or wihout BI-D, and virus production was measured after 48 h. Mock treated cells were transfected with control plasmid pBluescript-SK+. Average values with SD are shown (N = 3). C. The virus stock produced in (B) was used for infection of SupT1 T cells. No additional BI-D was added during culturing. The CA-p24 level in the culture medium was monitored by ELISA.

To test the impact of ALLINIs on HIV-1 RNA processes during virion assembly and maturation, we choose the compound BI-D, which inhibits the HIV-1 NL4-3 strain on SupT1 [16] and C8166 T cells [54]. To confirm the inhibitory effect, we infected SupT1 T cells with the HIV-1 LAI strain and cultured the cells with or without BI-D (700 nM, 5x EC_50_). Viral spread was monitored by measuring the CA-p24 level in the culture supernatant. Whereas efficient virus replication resulting in a rapid increase in CA-p24 level was scored in the control culture, HIV-1 LAI was efficiently blocked by BI-D (Fig 1A).

To test the effect of BI-D on virus production, we transfected 293T cells with the HIV-1 encoding plasmid pLAI [50] and cultured the cells in the presence or absence of BI-D. The virus containing supernatant was harvested 48 h later and the CA-p24 level was determined by ELISA. Virus production on 293T cells was not affected by BI-D, indicating that viral gene expression, including the processes of transcription and translation, were not inhibited (Fig 1B). Consistent with these results, virus production by PBMCs (determined by measuring the CA-p24 level in the culture medium) was previously shown not to be affected by BI-D [16]. Next, we tested whether virus produced by 293T cells in the presence of BI-D is infectious on SupT1 T cells. No additional BI-D was added during SupT1 culturing. Although we did not wash away BI-D that is present in the virus stock, the high dilution factor (∼2500x) and the subsequent passaging of the cell cultures every 3–4 days, makes a sustained antiviral effect unlikely. Efficient viral spread was observed upon infection of the cells with virus produced in the absence of BI-D. The presence of BI-D during virus production severely hampered virus infectivity such that no spreading infection could be established (Fig 1C). Similar effects have been reported for other ALLINIs [9], [10], [13], [14], [16].

### BI-D does not affect HIV-1 RNA packaging

**Figure 2:**
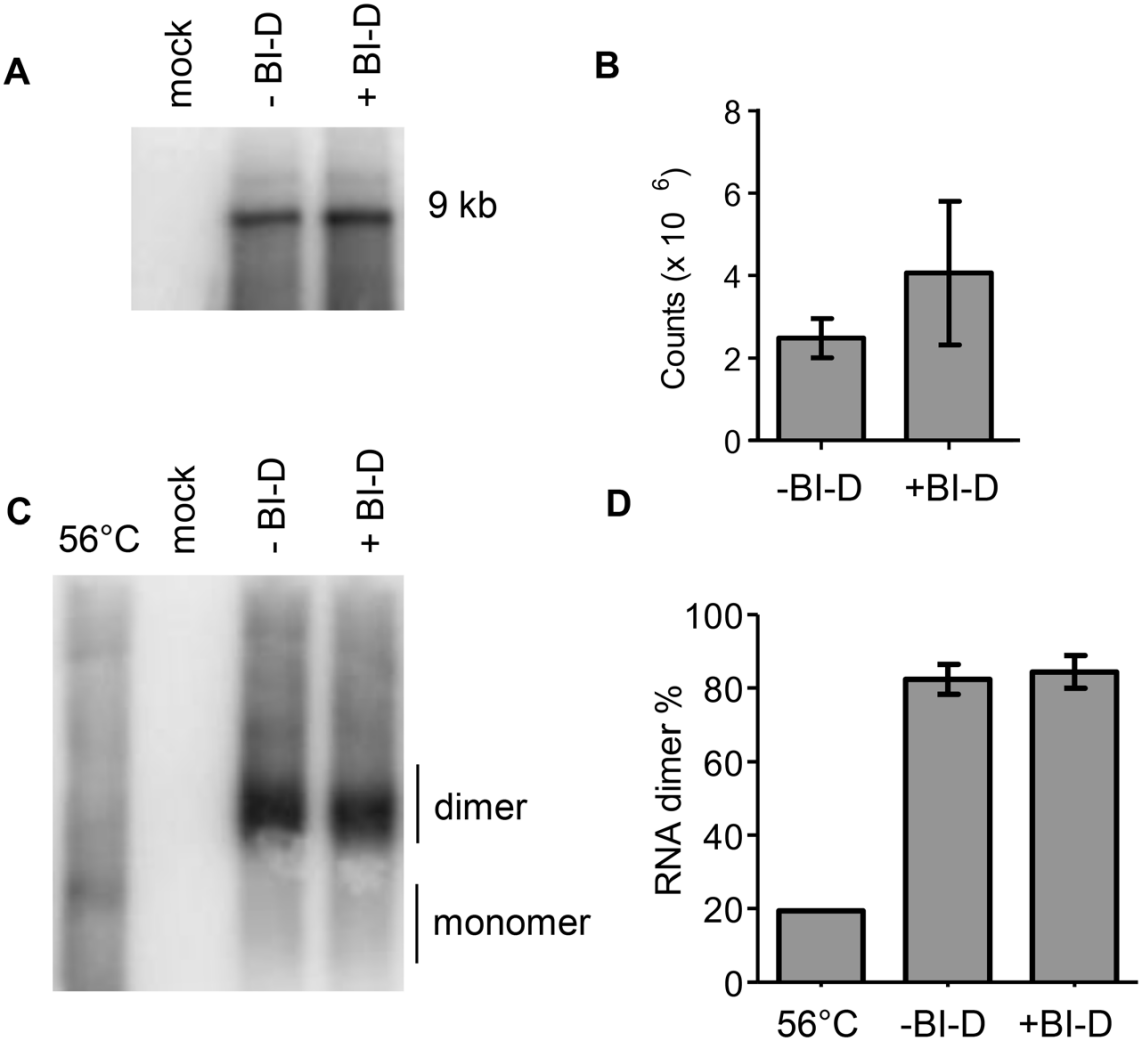
HIV-1 RNA packaging and dimerization. Viral RNA was isolated from virion particles produced in the presence or absence of BI-D. For mock samples, RNA was isolated from pBluescript-SK+ transfected cells. A. Virus RNA was analyzed on a denaturing northern blot to measure RNA packaging. The position of the full-length 9 kb viral genome is indicated. B. Quantification of the 9-kb viral RNA detected in panel A. The average value (N = 3) with SD is shown. C. Viral RNA was analyzed on a non-denaturing northern blot to analyze the dimerization status. The position of the dimer and monomer are indicated. To identify the position of the monomer, the – BI-D RNA sample was incubated at 56°C for 10 min before analysis. D. The monomer and dimer bands observed in panel C were quantified to measure the level of dimerization. The average value with SD is shown (N = 3).

Although the class of ALLINIs was originally designed to inhibit the early IN-LEDGF/p75 interaction, subsequent studies indicated that the major inhibitory effect is exerted during the late phase of HIV-1 virion assembly and maturation [10], [11], [15], [16]. One effect is the mislocalization of the RNP outside of the capsid core. We tested whether the HIV-1 RNA genome is packaged in these misformed virion particles. Virus was produced on 293T cells with or without BI-D. The virions were pelleted from the culture supernatant by ultracentrifugation and the viral RNA was subsequently extracted. The amount of full-length HIV-1 RNA was determined by denaturing northern blot analysis of equal amounts of virus particles (based on the CA-p24 level). A similar level of the full-length 9-kb viral RNA was observed in the presence and absence of BI-D, which shows that BI-D does not affect the packaging level of full-length genomic RNA (Fig 2A and bands were quantitated in Fig 2B). This result confirms earlier reports that used qPCR methods to quantify HIV-1 RNA [15], [16].

### HIV-1 RNA dimerization is not affected by BI-D treatment

As for all retroviruses, the HIV-1 genome is packaged as a non-covalently linked RNA dimer. It is thought that labile RNA dimers are packaged, which are subsequently stabilized by additional intermolecular interactions that occur during virus maturation (reviewed in [55]). The BI-D induced virus assembly defect could have an impact on HIV-1 RNA dimerization. We therefore investigated the dimeric state of the RNA genomes isolated from virus particles by running them on a non-denaturing gel, followed by northern blotting (Fig 2C and bands were quantitated in Fig 2D). Most of the genomic RNA extracted from untreated virions was in the dimeric state (∼82%). A similar level was observed for the BI-D treated virions.

**Figure 3:**
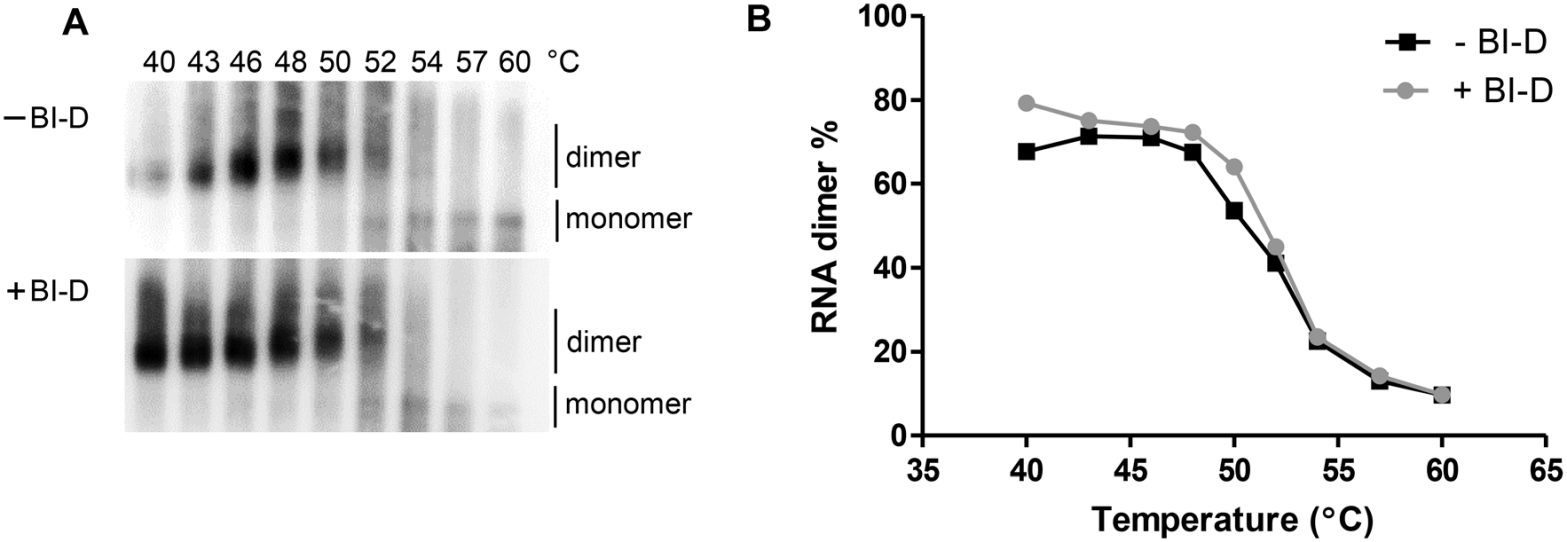
Thermal stability of HIV-1 RNA dimers from viruses cultured in the presence or absence of BI-D. A. Virion-derived RNA was heated for 10 min at increasing temperatures (40, 43, 46, 48, 50, 52, 54, 57 and 60°C) before analysis on a non-denaturing northern blot. The dimer and monomer positions are indicated. B. The monomer and dimer RNA bands observed in panel A were quantified to calculate the level of RNA dimerization at each temperature.

We next probed the thermostability of the RNA dimers as a measure of dimer maturation. The isolated viral RNA was incubated at increasing temperatures for 10 min before analysis on a non-denaturing gel followed by northern blotting (Fig 3A). Figure 3B shows the melting curves of HIV-1 dimeric RNA produced with or without BI-D. The melting temperature at which 50% of the dimer is converted into monomer was very similar for the virus produced in the presence and absence of BI-D (Tm = 51.9°C and 52.1°C, respectively). These combined results suggest that RNA dimer formation and maturation are normal in the presence of BI-D, despite the fact that the RNA genome is mislocalized outside the virus core.

### Virus produced in the presence of BI-D contains an active RT enzyme

During virus maturation, the RT enzyme is activated by release from the Gag-Pol polyprotein [30]. Jurado *et al.* showed that the process of reverse transcription of virus produced in the presence of BI-D is blocked [16]. One of the reasons could be improper Gag-Pol processing or the generation of an inactive RT enzyme due to the apparent virus maturation defect. Although normal amounts of RT enzyme were detected on western blot [16], we decided to extract the RT enzyme from virion particles and test its activity.

**Figure 4:**
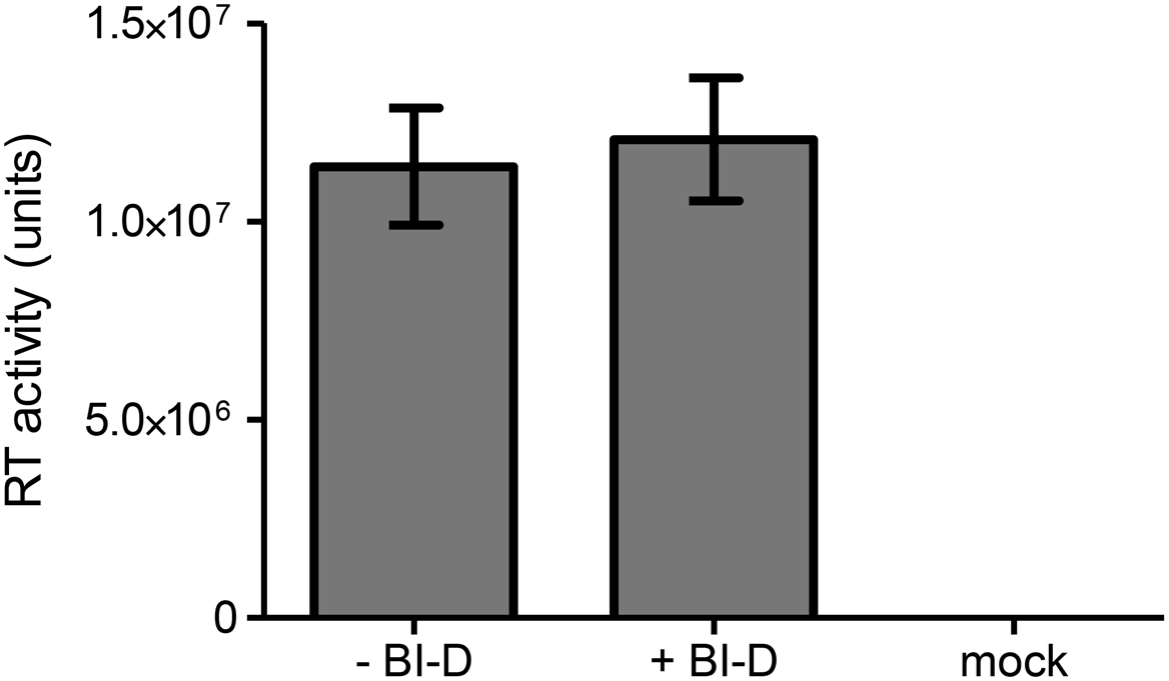
Activity of virus-extracted RT enzyme. Virus was produced with or without BI-D. Virus supernatant was incubated with an MS2 RNA template and dNTPs to reverse transcribe the template RNA. The activity of the RT enzyme was determined by quantitation of the cDNA product by qPCR. Serial dilutions of AMV-RT were used to generate a standard curve. Average values with SD are shown (N = 2). Mock: supernatant of cells transfected with control plasmid, pBluescript-SK+.

Virus was produced by 293T cells in the presence or absence of BI-D, yielding similar CA-p24 levels. The RT enzyme was extracted from virions in the culture supernatant by addition of Triton-X100 and used to reverse transcribe an MS2 RNA template with an annealed DNA primer upon addition of dNTPs. The cDNA product was quantified by real-time PCR (qPCR) using a MS2-specific probe. After correction for the virus input based on CA-p24, we measured a similar RT activity in the extracts of viruses generated with or without BI-D (Fig 4). This result indicates that BI-D does not affect RT production and activity.

### BI-D does not affect tRNA^lys3^ primer placement and activation

**Figure 5:**
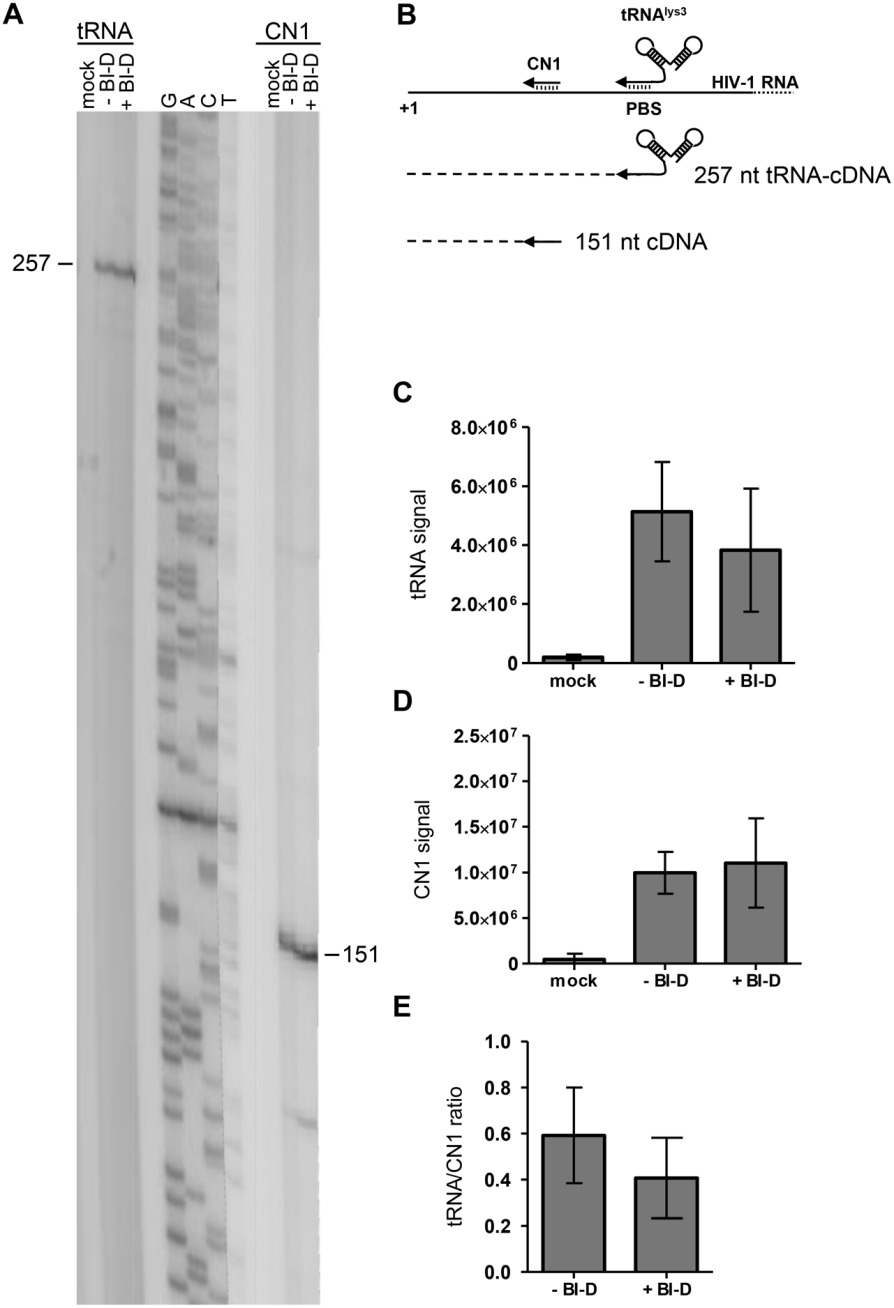
tRNA^lys3^ occupancy of the viral RNA. Viral RNA-tRNA^lys3^ complexes were isolated from virions produced in the presence or absence of BI-D and analyzed by primer extension. Extension of the natural tRNA^lys3^ primer bound to the PBS yields a product of 257 nt. Extension of a heat-annealed CN1 primer results in a product of 151 nt. A. Both tRNA^lys3^ and CN1 primers were extended by addition of exogenous HIV-1 RT (p66/51) enzyme and dNTPs. The resulting DNA products were run on a denaturing polyacrylamide gel. Lanes of one gel were merged. B. Schematic showing the primer extension assay. C. Quantification of the 257-nt band produced by the natural tRNA^lys3^ primer. D. Quantification of the 151-nt band produced by the DNA primer CN1. E. tRNA^lys3^ occupancy of the PBS was determined by calculating the tRNA to CN1 product ratio. The average value with SD is shown (N = 4). Mock: the RNA isolation procedure was performed on the supernatant of cells transfected with control plasmid pBluescript-SK+.

The BI-D impact on reverse transcription could also be caused by improper placement of the host tRNA^lys3^ primer on the viral RNA, which is the essential first step of reverse transcription. To visualize the tRNA^lys3^ primer bound to the viral RNA, tRNA^lys3^ can be extended *in vitro* by the incubation of the virion-extracted RNA with dNTPs and exogenous RT enzyme (Fig 5B) [56]. For this, viral RNA-tRNA^lys3^ complexes were isolated from virus particles produced by 293T cells in the presence or absence of BI-D. The HIV-1 RT enzyme and dNTPs were added to the RNA isolated from an equal amount of virions (as determined by the input CA-p24 level). The resulting 257-nt tRNA^lys3^-cDNA product was visualized on a denaturing polyacrylamide gel and quantified with a phosphor imager. A similar level of this product was observed with the RNA isolated from untreated or BI-D treated virions. This result shows that the primer is present and that it can be extended efficiently, irrespective of the presence of BI-D during virus production (Fig 5A, C). Extension of the heat-annealed control DNA primer CN1 yielded a similar level of 151-nt cDNA product for the BI-D treated and untreated virus sample (Fig 5A, D). Because the DNA primer CN1 was heat-annealed, the associated tRNA^lys3^ primer was removed, which explains why the tRNA^lys3^-derived cDNA product was not observed in these lanes. The similar level of CN1 product confirms that HIV-1 RNA was packaged at similar efficiency with or without BI-D (Fig 5A, D). The relative tRNA^lys3^/CN1 ratio was not affected either, which illustrates that the tRNA^lys3^ occupancy of the viral RNA was not affected by BI-D (Fig 5E). Very similar results were obtained by addition of the AMV RT enzyme, instead of HIV-1 RT (results not shown). These results demonstrate that the formation of a proper initiation complex (HIV-1 RNA with annealed tRNA^lys3^ primer) is not influenced by BI-D.

## Discussion

Allosteric IN inhibitors that disrupt the IN-LEDGF/p75 interaction do not only affect the ‘early’ process of HIV-1 integration but, unexpectedly, also the ‘late’ process of maturation. Because of the intricate link between virus maturation and several RNA-mediated processes, we investigated the effect of ALLINI BI-D, the first compound for which an altered virion morphology was reported, on RNA processes during virion assembly. The combinatorial effect of ALLINIs on early and late processes and thus possibly on RNA mechanisms could pose a combination therapy in a single drug. We confirm that BI-D, when present during virus production, renders the progeny virus non-infectious. However, we did not observe any effect on HIV-1 genomic RNA packaging and dimerization, tRNA^lys3^ placement on the viral RNA and activity of the RT enzyme. This excludes a further expansion of the BI-D inhibitory potential to the RNA processes during HIV-1 virion maturation.

Using two different methods, denaturing northern blot (Fig 2A, B) and primer extension analysis (Fig 5A, D), we measured efficient HIV-1 RNA packaging in the presence of BI-D. Using a quantitative RT-PCR approach, similar results were described for BI-D [16] and CX05045 [15]. We also could not detect differences in dimer formation of the HIV-1 RNA genome (Fig 2C, D) and the stability of these dimers (Fig 3). These results imply that RNA packaging and dimerization are not affected by its aberrant localization outside the virus core in BI-D treated virions. A similar phenomenon has been observed previously. Two studies used suboptimal levels of two PR inhibitors during the production of HIV-1 virus particles to monitor Gag and Gag-Pol processing, RNA dimerization and virus maturation [57], [58]. They observed an increase in immature and aberrant virus particles, but only a minor destabilization of the RNA dimer was measured at the highest inhibitor dose. It was suggested that RNA dimer maturation does not require a correct virus core conformation, which is confirmed in this study using the allosteric IN-inhibitor BI-D.

Cell entry is not affected for virus produced in the presence of several ALLINIs, but early reverse transcription products were severely reduced with compounds BI-D, CX05045 and GS-B [11], [15], [16]. We show that the packaged RT enzyme is fully active and that the tRNA^lys3^ primer is correctly placed on the genomic RNA for cDNA synthesis. Thus, all basic factors required for reverse transcription are functionally present in BI-D treated virions, which nevertheless are severely defective in reverse transcription. Multiple studies showed that the first step of reverse transcription (initiation and production of strong-stop cDNA) is already inhibited [11], [15], [16]. This may indicate that the components, although present and active in BI-D treated virions, are not located at the right position at the right time.

Current antiretroviral therapies use drugs that target viral enzymes (RT, IN, PR) or virus entry into the target cell. None of the current drugs exert an effect at the HIV-1 RNA level. With the unexpected late effect of ALLINIs on virus maturation, besides its inhibiting effect on integration, this class of drugs is remarkably pleiotropic, but BI-D does not interfere with the viral RNA processes.
